# Gestational age-specific sex difference in mortality and morbidities of preterm infants: A nationwide study

**DOI:** 10.1038/s41598-017-06490-8

**Published:** 2017-07-21

**Authors:** So-Yeon Shim, Su Jin Cho, Kyoung Ae Kong, Eun Ae Park

**Affiliations:** 1 Department of Pediatrics, School of Medicine, Ewha Womans University, Seoul, Korea; 2 Department of Preventive Medicine, School of Medicine, Ewha Womans University, Seoul, Korea

## Abstract

This study aims to determine whether male sex has adverse effect on mortality and morbidities in very low birth weight infants (VLBWI) <30 weeks of gestation and to ascertain this sex effect, stratified by gestational age, adjusting for perinatal risk factors. This is a population-based study from Korean Neonatal Network for VLBWI born at 23^+0^ and 29^+6^ weeks of gestation between January 2013 and December 2014. The primary outcome was gestation-specific sex difference in the occurrence of mortality, combined morbidities, and individual morbidity. A total of 2228 VLBWI were enrolled (males, 51.7%). Mortality was not different between sexes. The risk of bronchopulmonary dysplasia and combined morbidities was significantly higher in males ≤25 weeks of gestation (odds ratio [OR] 2.08, 95% confidence interval [CI] 1.35–3.20 and OR 2.00, CI 1.19–3.39, respectively). Males had a significantly higher incidence of periventricular leukomalacia at 23 and 29 weeks of gestation. The risk of severe retinopathy of prematurity was higher in females >25 weeks of gestation. Although both sexes have similar risk for mortality, male sex remains an independent risk for major morbidities, especially at ≤25 weeks of gestation. The risk of each outcome for males has a specific pattern with increasing gestational age.

## Introduction

Preterm birth is defined by the World Health Organization (WHO) as all births before 37 completed weeks of gestation^1^. Infants were divided according to birth weight into very low birth weight infants (VLBWIs; infants born with <1,500 g) and extremely low birth weight infants (infants born with <1,000 g)^2^. Approximately 15 million infants are born preterm each year, representing a preterm birth rate of 11.1%^3^. In Korea^4^, preterm birth accounts for approximately 8% of live birth, similar to East Asia (7.4%) and Western countries (8.6%)^3^. Complications of preterm birth are the single largest direct cause of neonatal deaths, responsible for 35% of the world’s 3.1 million deaths a year^5^, with VLBWI being a main cause of neonatal death and major complications^6^. To date, a number of guidelines for clinicians and parents on neonatal outcomes at various gestational ages have been developed^7–9^. Many factors are known to affect the mortality and morbidities of preterm infants. Among them, higher birth weight, higher gestational age, and the use of antenatal steroid have been consistently linked to better prognosis^10^. However, information on gestation-specific sex effect on outcomes is very limited^10–12^. Sex difference in clinical study is important because hormonal, physiological, and developmental differences between males and females could lead to sex-specific outcomes^13^. Previous studies have shown that the male sex is associated with an increased risk of mortality in preterm infants^10, 14–16^. Male infants are also associated with worse respiratory outcomes, such as respiratory distress syndrome and bronchopulmonary dysplasia (BPD), as well as intraventricular hemorrhage (IVH) and retinopathy of prematurity (ROP)^10, 13, 17–20^. Later surfactant production and higher density of androgen receptors in male fetus contribute to increased alveolar resistance and the modulation of bronchiole budding in the early fetal lung^21–23^, and these effects may continue and worsen the respiratory outcome in males after birth. Recently, a few studies have not found any difference in sex and mortality, due to progress in neonatal intensive care^11, 24^. Although gestational age is the most powerful factor for neonatal outcomes, only a few studies showed the sex effects, adjusted by gestational age^10, 12^. Furthermore, as far as we know, there were no sex studies that considered various perinatal factors including antenatal steroid use, maternal diabetes, chorioamnionitis, intrauterine growth restriction (IUGR), and multiple births in preterm infants.

The present study is a retrospective observational study based on the Korean Neonatal Network (KNN), which is a nationwide database on VLBWI across South Korea. The aim of the present study was to investigate whether male sex has disadvantage on mortality and short-term morbidities in preterm infants. Because mortality and major morbidities frequently occur at gestational age ≤29 weeks, with lower risk from 30 weeks onward^24–26^, we focused on VLBWIs born at <30 weeks of gestation. We aimed to ascertain sex effect, stratified by gestational age in this population, adjusting for perinatal risk factors that may affect neonatal outcomes. Furthermore, because previous studies have reported that preterm infants born at ≤25 weeks of gestation showed especially higher mortality and morbidities^9, 11, 25, 27, 28^, we performed a subgroup analysis according to gestational age group, from 23 to 25 weeks of gestation and from 26 to 29 weeks of gestation.

## Results

### Population

**Figure 1:**
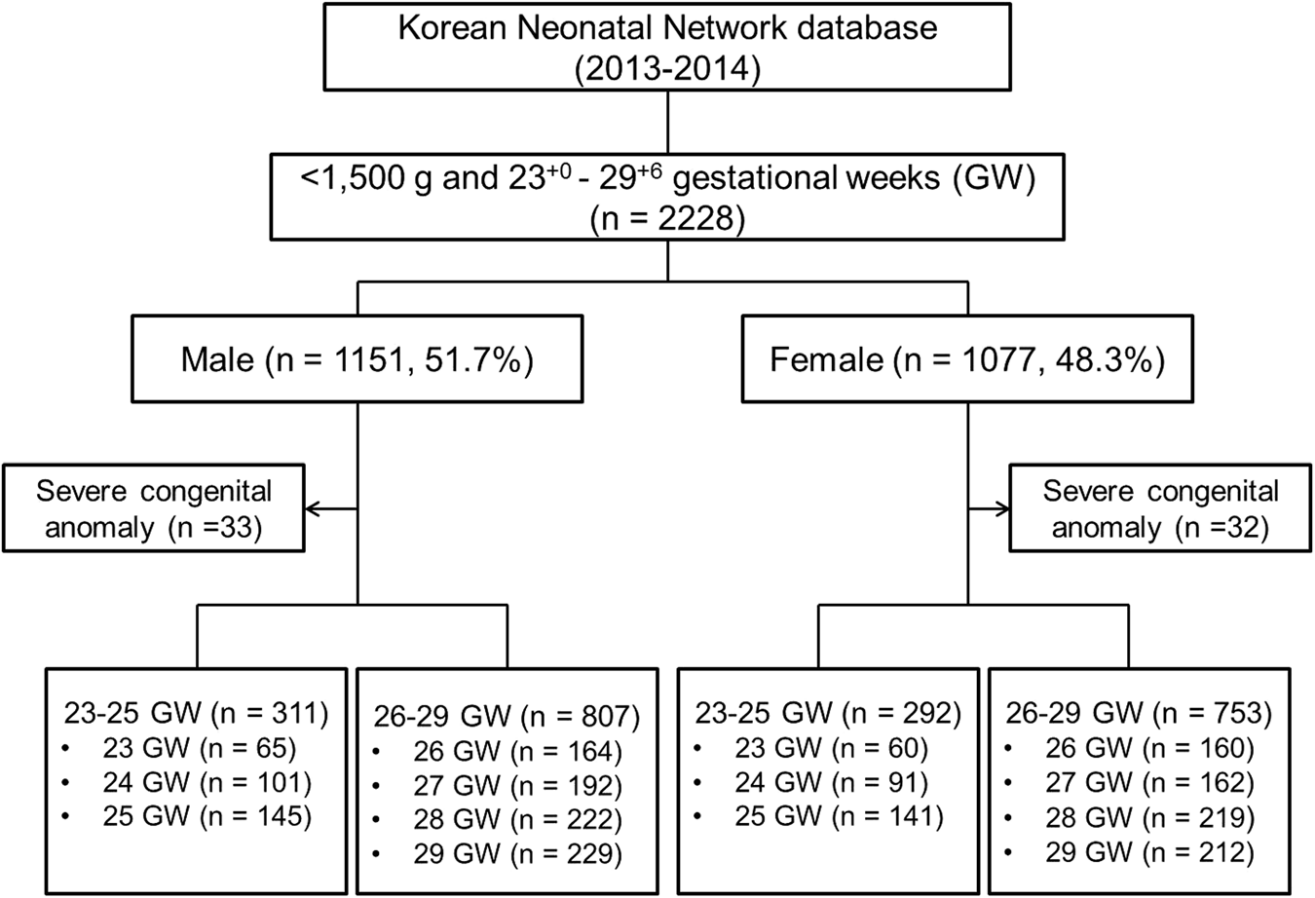
Study population from the Korean Neonatal Network database.

A total of 2228 VLBWIs (males, n = 1151, 51.7%; females, n = 1077, 41.3%) with gestational age of <30 weeks were registered to the KNN during the study period. Severe congenital anomaly was noted in 65 infants (33 males and 32 females). In male infants, there were 6 congenital heart diseases, 4 genitourinary tract defects, 2 central nervous system anomalies, 12 digestive organ anomalies, 1 pulmonary abnormality, 2 chromosomal anomalies, and 5 other anomalies. In female infants, there were 12 congenital heart diseases, 1 genitourinary tract defect, 2 central nervous system anomalies, 10 digestive organ anomalies, 1 pulmonary abnormality, 1 chromosomal anomaly, and 5 other anomalies. The number of male and female infants in the two gestational age groups and at individual gestational age is shown in Fig. 1.

### Infants’ characteristics

There was no significant sex difference in gestational age, delivery mode, outborn status, Apgar scores, length of hospital stay, and duration on ventilation. The rate of IUGR was significantly higher in female infants (*P* = 0.026), but in the subgroup analysis, this significance disappeared. Overall, male infants had higher birth weight (*P* < 0.001), height (*P* < 0.001), and head circumference (*P* = 0.001) at birth and at discharge (all *P*s < 0.001). In the subgroup analysis, the 23–25 weeks of gestation group showed that male infants had higher weight and head circumference at birth (*P* < 0.001 and *P* = 0.002, respectively) and at discharge (all *P*s = 0.028). In the 26–29 weeks of gestation group, male infants had higher birth weight, height, and head circumference at birth (all *P*s < 0.001) and at discharge (*P* = 0.009, *P* = 0.007, and *P* = 0.018, respectively) (Table 1).

**Table 1:** Infants’ characteristics and sex difference according to gestational age group.

Variables	Total enrolled infants			23–25 weeks of gestation group			26–29 weeks of gestation group		
	Male (n = 1118)	Female (n = 1045)	*P*	Male (n = 311)	Female (n = 292)	*P*	Male (n = 807)	Female (n = 753)	*P*
Gestational age (weeks)	27^+1^ (2^+0^)	27^+1^ (2^+0^)	0.971	24^+5^ (0^+2^)	24^+5^ (0^+2^)	0.752	28^+0^ (1^+2^)	28^+0^ (1^+2^)	0.971
Cesarean section	773 (69.1)	738 (70.6)	0.453	211 (67.8)	190 (65.1)	0.470	562 (69.6)	548 (72.8)	0.172
Outborn	47 (4.2)	37 (3.5)	0.425	10 (3.2)	11 (3.8)	0.712	37 (4.6)	26 (3.5)	0.256
Intrauterine growth restriction	63 (5.6)	84 (8.0)	0.026	19 (6.1)	26 (8.9)	0.192	44 (5.5)	58 (7.7)	0.072
Birth weight (g)	1009 (261)	946 (259)	<0.001	746 (136)	700 (137)	<0.001	1111 (224)	1041 (230)	<0.001
Height at birth (cm)	35.5 (3.4)	34.8 (3.4)	<0.001	32.2 (2.3)	31.8 (2.2)	0.087	36.6 (3.0)	35.8 (3.1)	<0.001
Head circumference at birth (cm)	25.1 (2.2)	24.5 (2.2)	0.001	22.8 (1.3)	22.4 (1.6)	0.002	25.8 (1.9)	25.2 (1.8)	<0.001
Weight at discharge (g)	2553 (954)	2418 (907)	<0.001	2384 (1287)	2164 (1154)	0.028	2619 (781)	2516 (770)	0.009
Height at discharge (cm)	45.0 (5.1)	44.2 (5.4)	<0.001	43.7 (6.6)	42.5 (7.0)	0.057	45.4 (4.3)	44.8 (4.5)	0.007
Head circumference at discharge (cm)	32.1 (3.3)	31.6 (3.5)	<0.001	31.4 (4.6)	30.5 (4.8)	0.028	32.3 (2.7)	32.0 (2.8)	0.018
Apgar scores 1 min	3.3 (1.6)	3.4 (1.8)	0.278	3.2 (1.6)	3.1 (1.7)	0.413	4.4 (1.9)	4.6 (1.8)	0.070
Apgar scores 5 min	4.1 (1.9)	4.1 (1.9)	0.775	5.6 (1.8)	5.3 (2.0)	0.064	6.7 (1.7)	6.8 (1.6)	0.292
Length of stay (days)	76 (44)	76 (43)	0.939	88 (62)	84 (57)	0.383	72 (33)	74 (37)	0.306
Duration on ventilation (days)	43.8 (39.7)	41.9 (38.8)	0.241	63.2 (50.1)	52.5 (43.0)	0.133	36.4 (31.8)	35.8 (35.3)	0.736

Data are presented as n (%) or mean (SD).

### Maternal characteristics

The prevalence of maternal diabetes, histologic chorioamnionitis, multiple births, and the use of antenatal steroid were not different between sexes (Table 2). The mothers of the female infants in the 26–29 weeks of gestation group had a higher rate of maternal hypertension (*P* = 0.044).

**Table 2:** Infants’ sex difference in maternal records according to gestational age group.

Variables	Total study population			23–25 weeks of gestation group			26–29 weeks of gestation group		
	Male (n = 1118)	Female (n = 1045)	*P*	Male (n = 311)	Female (n = 292)	*P*	Male (n = 807)	Female (n = 753)	*P*
Multiple births	367 (32.8)	336 (32.2)	0.738	126 (40.5)	101 (34.6)	0.133	241 (29.9)	235 (31.2)	0.564
Maternal diabetes	98 (8.8)	73 (7.0)	0.125	17 (5.5)	14 (4.8)	0.709	81 (10.0)	59 (7.8)	0.709
Maternal hypertension	117 (10.5)	139 (13.3)	0.041	17 (5.5)	19 (6.5)	0.590	100 (12.4)	120 (15.9)	0.044
Chorioamnionitis	386 (34.5)	351 (33.6)	0.646	151 (48.6)	129 (44.2)	0.282	235 (29.1)	222 (29.5)	0.875
Antenatal steroid	881 (78.8)	825 (78.9)	0.934	235 (75.6)	217 (74.3)	0.724	646 (80.0)	608 (80.7)	0.730

Data are presented as n (%).

### Mortality and morbidities

Mortality was not different between male and female infants. In all enrolled infants, males had a higher prevalence of combined morbidity (*P* = 0.011). Males also showed a higher occurrence of BPD (*P* = 0.012), IVH grade ≥3 (*P* = 0.049), and cystic periventricular leukomalacia (PVL) (*P* = 0.014) (Table 3). In the analysis according to individual gestational age, the odds ratio (OR) of the male versus female sex for combined morbidities was the highest at 23 weeks of gestation (OR 5.0, 95% confidence interval [CI] 1.1–55.7, *P* = 0.038). This OR tended to decrease and then lost significance from 26 weeks of gestation. BPD showed a similar pattern; the OR of males on BPD was significant at 24 and 25 weeks of gestation (OR 2.7, 95% CI 1.2–6.3, *P* = 0.020 and OR 1.9, 95% CI 1.1–3.3, *P* = 0.004, respectively), and this significance disappeared from 26 weeks of gestation. Males had a significantly higher incidence of PVL at 23 and 29 weeks of gestation (OR 4.0, 95% CI 1.1–15.5, *P* = 0.042 and OR 2.4, 95% CI 1.1–5.0, *P* = 0.026, respectively). In contrast, males had a lower incidence of ROP at 26 and 27 weeks of gestation (OR 0.5, 95% CI 0.3–0.9, *P* = 0.026 and OR 0.4, 95% CI 0.2–0.9, *P* = 0.022, respectively). There was no significant sex difference in IVH grade ≥3 and necrotizing enterocolitis (NEC) at any gestation age (Table 4).

**Table 3:** Sex difference for the occurrence of mortality and the prevalence of major neonatal morbidities, stratified by gestational age group.

Variables	Subgroup	Male (n, %)	Female (n, %)	*P* (*χ* ^*2*^)	Adjusted OR* (95% CI)	P*
Mortality	Total	179/1118 (16.0)	165/1045 (15.8)	0.888	1.04 (0.82–1.31)	0.756
	23–25 wk	108/311 (34.7)	96/292 (32.9)	0.631	1.13 (0.79–1.59)	0.508
	26–29 wk	71/807 (8.8)	69/753 (9.2)	0.801	0.97 (0.68–1.39)	0.872
BPD	Total	419/951 (44.1)	346/888 (39.0)	0.027	1.28 (1.06–1.54)	0.012
	23–25 wk	155/210 (73.8)	113/196 (57.7)	0.001	2.08 (1.35–3.20)	0.001
	26–29 wk	264/741 (35.6)	233/692 (33.7)	0.437	1.12 (0.90–1.40)	0.308
IVH, grade ≥ 3	Total	176/1082 (16.3)	130/1002 (13.0)	0.034	1.28 (1.01–1.65)	0.049
	23–25 wk	99/292 (33.9)	73/268 (27.2)	0.088	1.38 (0.95–2.01)	0.096
	26–29 wk	77/790 (9.7)	57/734 (7.8)	0.172	1.27 (0.88–1.84)	0.196
PVL	Total	140/1073 (13.0)	95/994 (9.6)	0.013	1.42 (1.07–1.87)	0.014
	23–25 wk	47/285 (16.5)	33/262 (12.6)	0.198	1.27 (0.78–2.08)	0.334
	26–29 wk	93/788 (11.8)	62/732 (8.5)	0.032	1.47 (1.04–2.06)	0.028
ROP, grade ≥ 3	Total	159/952 (16.7)	170/902 (18.8)	0.227	0.89 (0.70–1.13)	0.343
	23–25 wk	105/209 (50.2)	84/202 (41.6)	0.078	1.37 (0.92–2.04)	0.123
	26–29 wk	54/743 (7.3)	86/700 (12.3)	0.001	0.59 (0.41–0.85)	0.004
NEC	Total	109/1116 (9.8)	81/1039 (7.8)	0.107	1.34 (0.99–1.81)	0.062
	23–25 wk	52/311 (16.7)	41/290 (14.1)	0.382	1.27 (0.80–2.01)	0.305
	26–29 wk	57/805 (7.1)	40/749 (5.3)	0.156	1.44 (0.94–2.20)	0.094
Combined morbidities	Total	521/941 (55.4)	444/882 (50.3)	0.032	1.27 (1.06–1.54)	0.011
	23–25 wk	179/208 (86.1)	143/191 (74.9)	0.005	2.00 (1.19–3.39)	0.009
	26–29 wk	342/733 (46.7)	301/691 (43.6)	0.240	1.18 (0.95–1.46)	0.128

*Values are odds ratio and 95% confidence interval. Values are adjusted by gestational age, histologic chorioamnionitis, intrauterine growth restriction, maternal diabetes, maternal hypertension, multiple births, delivery mode, and the use of antenatal steroid.

**Table 4:** Sex difference for the occurrence of mortality and the prevalence of major neonatal morbidities, stratified by individual gestational age.

GA (weeks)	Mortality		BPD		IVH, grade ≥ 3		PVL		ROP, grade ≥ 3		NEC		Combined morbidities	
	OR* (95% CI)	*P*	OR* (95% CI)	*P*	OR* (95% CI)	*P*	OR* (95% CI)	*P*	OR* (95% CI)	*P*	OR* (95% CI)	*P*	OR* (95% CI)	*P*
23	0.93 (0.44–2.07)	0.821	2.86 (0.57–14.54)	0.266	2.41 (0.97–5.30)	0.057	4.05 (1.06–15.5)	0.042	2.90 (0.83–10.02)	0.082	1.98 (0.73–5.40)	0.121	5.00 (1.10–55.67)	0.038
24	1.15 (0.62–2.15)	0.993	2.72 (1.18–6.25)	0.020	1.50 (0.77–2.66)	0.183	1.33 (0.50–3.54)	0.538	1.36 (0.65–2.86)	0.298	0.97 (0.61–2.13)	0.959	1.68 (0.58–4.90)	0.383
25	1.14 (0.63–2.04)	0.423	1.86 (1.05–3.29)	0.004	1.04 (0.58–1.93)	0.721	0.98 (0.49–1.93)	0.802	1.20 (0.68–2.11)	0.333	1.34 (0.64–2.75)	0.854	2.09 (1.07–4.09)	0.012
26	1.11 (0.62–1.97)	0.781	1.26 (0.77–2.05)	0.315	0.884 (0.47–1.65)	0.657	1.29 (0.67–2.48)	0.193	0.50 (0.27–0.92)	0.026	1.20 (0.56–2.57)	0.580	1.20 (0.71–2.03)	0.640
27	1.03 (0.48–2.23)	0.634	1.06 (0.67–1.68)	0.951	1.72 (0.84–3.53)	0.109	1.31 (0.64–2.68)	0.562	0.41 (0.19–0.88)	0.022	1.79 (0.83–3.89)	0.132	1.06 (0.67–1.67)	0.966
28	0.81 (0.35–1.86)	0.368	1.32 (0.86–2.03)	0.272	1.06 (0.46–2.46)	0.782	1.40 (0.70–2.80)	0.238	1.07 (0.49–2.31)	0.936	1.83 (0.70–4.76)	0.165	1.48 (0.99–2.23)	0.239
29	1.08 (0.39–3.00)	0.406	1.01 (0.61–1.65)	0.845	2.44 (0.74–8.05)	0.267	2.36 (1.11–5.02)	0.026	0.44 (0.13–1.54)	0.134	1.01 (0.27–4.00)	0.892	0.99 (0.64–1.55)	0.929

*Values are adjusted odds ratio and 95% confidence interval. Values are adjusted by histologic chorioamnionitis, intrauterine growth restriction, maternal diabetes, maternal hypertension, multiple births, delivery mode, and the use of antenatal steroid according to individual gestational age (GA).

Subgroup analysis demonstrated that male infants ≤25 weeks of gestation had a higher incidence of combined morbidities (OR 2.0, 95% CI 1.2–3.4, *P* = 0.009) and BPD (OR 2.1, 95% CI 1.4–3.2, *P* = 0.001). In the 26–29 weeks of gestation group, female infants had a higher incidence of ROP (OR 0.6, 95% CI 0.4–0.9, *P* = 0.004), and male infants had a higher incidence of PVL (OR 1.5, 95% CI 1.0–2.1, *P* = 0.028) (Table 3).

## Discussion

In the present study, male VLBWIs born <30 weeks of gestation had similar incidence of mortality compared with female infants but had higher prevalence of combined morbidities and higher occurrence of BPD, severe IVH, and PVL. In contrast to previous studies^15, 16, 29^, the present study performed subgroup analysis according to gestational age and could suggest that male infants have a higher risk for BPD and combined morbidities under 25 weeks of gestation. Furthermore, male infants present specific pattern of risk for each outcome with increasing gestational age.

To date, many studies have confirmed a survival advantage for female infants compared with male infants^10, 14–16, 30^. Only a few studies did not find any difference in mortality between sexes^11, 24^; a Canadian cohort study^11^ suggested that there was no significant sex difference in mortality at >24 weeks of gestation, but survival was higher in female infants born at 24 weeks of gestation. Ray and Platt^24^ reported no effect of sex on mortality either for all gestations or among infants at 23–25 weeks of gestation. Our finding of no sex difference in mortality adds weight to those studies. Several possible reasons can be considered. The use of antenatal steroids and exogenous surfactant may have improved survival rate, more notably in male than in female infants. The KNN registry does not include information on stillbirth after onset of labor or delivery room death of live-born infants. This might affect our mortality results because the male disadvantage for adverse outcomes commences from early pregnancy^31^. Our data showed that female infants have significantly lower birth weight compared with male infants at the same gestational age. This finding was also observed in other studies^10, 12, 32^, and recently, a preterm growth chart for each sex has been provided^33^. Many previous studies that reported sex difference were stratified by birth weight^14, 15^, which might lead to the comparison of more mature female infants with less mature male infants, resulting in female advantage for survival. However, not all sex differences were eliminated in the present study, and female infants continue to have lower incidences of major morbidities. Previous studies have shown increased risk for BPD and IVH in male infants born preterm^10, 13, 20^. The present study analyzed sex difference, stratified by gestational age, and we found that the risk for combined morbidities and BPD was higher in male infants with ≤25 weeks of gestation compared with female infants of same gestational age; the OR of males gradually decreased with increasing gestational age and then lost significance from 26 weeks of gestation. Severe ROP has a similar pattern; however, it showed opposite OR results as of 25 weeks of gestation. These patterns suggest that 25 weeks of gestation seems to be a divided time point of male disadvantage for adverse neonatal outcomes. The subgroup analysis supported it with more detailed data – male infants in the 23–25 weeks of gestation group demonstrated a doubling of risk for combined morbidities and BPD, whereas the 26–29 weeks of gestation group showed a similar risk between sexes. For severe IVH, PVL, and NEC, male infants had generally higher risk throughout gestations compared with female infants, in terms of OR. The sex benefit of females is multifactorial and has an effect on prenatal and postnatal development^13, 19, 34^. To date, several animal studies had suggested possible pathologies for female advantage in neonatal outcomes. A different hormonal milieu in females is associated with increased organ maturation, compared with males^35^. In mice, males are known to have a higher arrest in alveolization and pulmonary angiogenesis and more inflammation compared with females^13^. Better antioxidant defense mechanism in female, including higher expression of glutathione peroxidase and superoxide dismutase^36, 37^, might contribute to female advantage in lung or brain injury in the perinatal period. A study reported that after resuscitation, the blood-brain barrier is better preserved and neuronal injury is lower in female piglets compared with male piglets^19^. These results could explain the increased incidence of BPD, IVH, or PVL in male infants of the present study but could not explain the gestation-specific sex effect on major outcomes. Until now, studies for gestation specific-sex difference are very limited^10, 12, 38^. Binet *et al*.^10^ reported the sex effect on short-term outcomes of extremely premature infants (≤27 weeks of gestation) born in Canada and found that the prevalence of BPD for infants born between 24 and 26 weeks were higher in males. Although their study is different from ours in study population and statistics, their finding is generally consistent with the present study. Ito *et al*.^38^ stated that male sex was a disadvantage for BPD in preterm infants at ≥26 weeks of gestation by using a 10-year database from the neonatal research network in Japan. As the medical situations and the study period were different in this database, the results seen were different from those in our study. In contrast to Binet *et al*.^10^ and Ito *et al*.^38^, the present study adjusted the perinatal factors that might affect neonatal outcomes and demonstrated that male sex is an independent risk factor of major morbidities, especially in infants at ≤25 weeks of gestation. Interestingly, in contrast to previous studies^17, 39, 40^, the present study found that severe ROP occurred more often in female infants after 26 weeks of gestation, and this remains after adjusting for perinatal risk factors including IUGR, maternal hypertension, use of antenatal steroid, and so on. At present, the explanation for the better outcome of ROP in male infants at this gestational period is unclear. Insulin-like growth factor 1 (IGF-1) is critical to normal vascular development, and the duration of low IGF-1 concentrations is strongly correlated with severity of ROP^41, 42^. Additional investigations for IGF-1 and sex according to gestations are warranted.

The strength of our study is that it is based on a large, geographical cohort, including approximately 70% of VLBWI in South Korea; therefore, the present study has greater relevance than a single-centered study. Although the present study was a retrospective study, data collection was done prospectively using the same strict guidelines of the KNN. However, we could not evaluate the long-term neurological outcome. Previous studies have demonstrated a correlation between short-term morbidities and long-term neurological outcomes^43, 44^.

## Conclusion

Male VLBWIs born at <30 weeks of gestation have similar incidence of mortality compared with female infants, which remains after adjusting for perinatal risk factors that may affect neonatal outcomes. However, male infants showed increased risk for combined morbidity, BPD, severe IVH, and PVL. Each outcome for males has a specific risk pattern with increasing gestational age. Subgroup analysis showed that the risk for combined morbidities and BPD is higher in males ≤25 weeks of gestation. In contrast, severe ROP occurred more often in females >25 weeks of gestation. Our results showed that sex difference, stratified by gestational age, is a major factor for neonatal outcomes of preterm infants. A guideline on neonatal outcomes that includes male disadvantage, especially for BPD and combined morbidities in infants ≤25 weeks of gestation, will help clinicians’ understanding and parents’ education.

## Methods

This is a population-based study of VLBWIs born 23^+0^ and 29^+6^ weeks of gestation between January 2013 and December 2014 and admitted to a neonatal intensive care unit (NICU) participating in the KNN^6^. Infants with severe congenital anomaly were excluded. The KNN is a nationwide database on VLBWI from 69 hospitals across South Korea. VLBWIs in these hospitals accounted for approximately 70% of VLBWIs in South Korea. Data were collected at each participating center and entered into a data program, and the study variables were defined according to the KNN manual. The present study was approved by the KNN data management committee.

### Ethics statement

The KNN registry was approved by the institutional review board (IRB) at each participating hospital, and informed consent was obtained from the parents at enrollment by the NICUs participating in the KNN. All methods were carried out in accordance with the IRB-approved protocol and in compliance with relevant guidelines and regulations.

### Primary outcome

The primary outcome was gestation-specific sex difference in the occurrence of mortality and the prevalence of combined morbidities among five outcomes and individual morbidity.

To identify gestation-specific sex outcomes, outcomes were tested at individual gestational age from 23^+0^ to 29^+6^ weeks of gestation. Furthermore, we divided the enrolled infants into two gestational age groups: the 23^+0^–25^+6^ weeks of gestation group and the 26^+0^–29^+6^ weeks of gestation group.

### Data collection

The infants’ data were collected from the KNN database (Fig. 1). Sex, gestational age, anthropometry measurements (including weight, height, and head circumference at birth and at discharge), delivery mode, Apgar score, outborn status, and IUGR were included. Length of hospital stay and duration on mechanical ventilation were also reviewed. Maternal data including multiple births, diabetes, hypertension, histological chorioamnionitis, and use of antenatal steroid were recorded. The following outcomes were collected for each sex: mortality during NICU hospitalization, BPD, IVH grade ≥3, PVL, ROP grade ≥3, and NEC.

### Definitions

VLBWI was defined infants born <1,500 g according to the WHO^2^. BPD was defined as a requirement for supplemental oxygen at 36 weeks of postmenstrual age^45^. IVH was defined using Papile’s criteria by cranial ultrasonography^46^. Because imaging was conducted using the individual policy of each hospital, the worst grade among several cranial ultrasonography results during hospitalization was adopted. PVL included only cystic PVL revealed by cranial ultrasonography or magnetic resonance imaging which was performed during hospitalization. NEC was defined according to Bell’s criteria (stage 2 or higher)^47^. ROP was defined according to the international classification of ROP^48^, and the maximum stage of ROP was adopted. To investigate the prevalence of combined morbidities, the present study developed the variable ‘combined morbidities’, which was defined as having one and more morbidities among the five morbidities. To develop the variable of combined morbidities, the value of each morbidity (0: no present, 1: present) was added, and the ‘0’ value in the sum was use to designate no combined morbidities. IUGR was defined as birth weight less than the 10th percentile for the gestational age based on a sex-specific growth chart^33^.

### Statistical analysis

Statistical analyses were performed using SPSS. Data are expressed as number (%) with OR and 95% CI or mean (standard deviation). Univariate analyses of categorical variables were performed by *χ* ^2^ test, and *t* test was used for continuous variables. Adjusted sex difference in mortality and morbidities was tested by multiple logistic regression. Multiple logistic regression was performed at individual gestational age and then for the two gestational age groups. Criteria for entry and removal were *P* < 0.05 and *P* > 0.10, respectively. The fit of models was checked with the Hosmer-Lemeshow goodness-of-fit test^49^. A *P* value < 0.05 with two-tailed comparisons was considered significant.

### Data Availability

The datasets analyzed during the current study are not publicly available due to the policy of Research of Korea Centers for Disease Control and Prevention but are available from the corresponding author on reasonable request.
